# Pulmonary function in patients with spinal deformity: have we been ignorant?

**DOI:** 10.1080/17453674.2020.1786267

**Published:** 2020-07-03

**Authors:** Miranda L Van Hooff, Niek Te Hennepe, Marinus De Kleuver

**Affiliations:** a Department of Orthopedics, Radboud University Medical Center, Nijmegen, The Netherlands;; b Department Research, Sint Maartenskliniek, Nijmegen, The Netherlands

Spine deformity refers to a broad spectrum of abnormal spinal curvatures, which are prevalent in all ages, and are seen by family physicians, orthopedists, and spine specialists. Pulmonary symptoms such as shortness of breath with exertion and reduced exercise tolerance are commonly experienced in both adolescents and adults with a spinal deformity. As yet, these clinically relevant pulmonary symptoms are not routinely monitored and may have health implications later in the patient’s life as pulmonary function gradually deteriorates with age. This Perspective aims to create awareness among care providers and researchers: attention should be paid to this underexposed domain when consulting with patients who have a spinal deformity, and adequate measurement instruments need to be developed to ultimately enhance the quality of care delivered to these patients.

## Adolescent idiopathic scoliosis (AIS)

In adolescence, idiopathic scoliosis is the most common type of scoliosis, occurring in about 2–3% of adolescents aged 16 years or younger (Weinstein et al. 2008). Various symptoms, such as back pain, reduced self-image, physical disability, and cardiopulmonary compromise, are well reported in AIS (Koumbourlis 2006, Weinstein et al. 2008). The effect of AIS on pulmonary function has been recognized and could lead, when untreated, to disability secondary to pulmonary symptoms, such as shortness of breath during daily functioning or exercise intolerance (Weinstein et al. 1981, Tsiligiannis and Grivas 2012, Weinstein 2019). Furthermore, it seems an important contributing factor to avoidance of activities and exercise training in patients with AIS (Lenke et al. 2002). Surprisingly, however, it is not routinely quantified/measured, and in all scientific publications this domain is rarely reported and extremely underexposed. Have we been ignorant?

In research, attempts have been made to objectively quantify pulmonary function in patients with AIS using clinical pulmonary function tests (PFTs). Even though a decrease in values of total lung capacity may be seen, dissociation exists between the measured pulmonary deficits and symptoms experienced by the patients (Tsiligiannis and Grivas 2012). Although PFTs are valuable to investigate and monitor patients with suspected or known respiratory pathology and to evaluate patients prior to major surgery (Ranu et al. 2011), conflicting evidence exists regarding their clinical value for both clinicians and patients in routine care for AIS patients. As such, obtaining routine PFTs for long-term patient surveillance and/or quantifying treatment effects is not standard practice as they lack clinical relevance, and are time consuming and costly to obtain.

## Adult spinal deformity (ASD)

Symptomatic ASD refers to various degenerative, progressive conditions and affects the thoracic or thoracolumbar spine throughout the aging process (Diebo et al. 2019). In younger adults the most common spinal deformity is persistent idiopathic scoliosis, whereas in middle-aged and older adults de novo degenerative lumbar scoliosis or adult degenerative scoliosis are more common (Silva and Lenke 2010, Diebo et al. 2019). Given its prevalence, with rapid increases expected over the coming decades, the disorder is of growing interest in health care. Global disparities in both assessment and treatment of ASD exist, resulting in high costs for society (Diebo et al. 2019).

Despite the (limited) knowledge regarding pulmonary function in adolescents, even less evidence is available regarding the effects of ASD on pulmonary function and the impact of (surgical) interventions for ASD on pulmonary function (Lehmann et al. 2015). A natural decline in pulmonary function is seen with aging but seems more pronounced in patients with untreated spinal deformity (Weinstein et al. 1981). Surgery in ASD patients has been reported to result in a significant deterioration in (clinical) PFTs two years following surgical correction (Lehmann et al. 2015). However, here too it is not routinely quantified/measured, and in scientific publications this domain is rarely reported and extremely underexposed.

## Pulmonary function: clinicians’ and patients’ perspective

### Clinicians’ perspective

Recently, for both AIS (De Kleuver et al. 2017) and ASD (Faraj et al. 2019) a standard outcome set was developed. The relevance of routinely assessing pulmonary function in both AIS and ASD was recognized by a worldwide group of expert clinicians, but such a patient-relevant measure is not currently available.

### Patients’ perspective

To obtain a first impression of pulmonary problems as experienced by patients with AIS and ASD, an anonymous exploratory survey was performed during an information day for

adolescents and adults with scoliosis in the Netherlands. Questions were related to pulmonary symptoms (including description in own words); limitations in daily functioning due to pulmonary symptoms; worsening of symptoms with increased fatigue; differences during the course of the day (answer options yes/no). Patient characteristics included age, concomitant respiratory disease, and previous surgical treatment for AIS or ASD. When previous spine surgery had been performed it was asked whether symptoms were different after surgery (relevant difference in terms of improvement and worsening, or no difference). After brief instruction, in total 58 patients completed the survey, aged 43 years (SD 12; categorized 10–17 years [n = 9]; 18–24 years [n = 10]; 25–40 years [n = 6], and ≥ 40 years [n = 33]). 3 patients reported having a respiratory disease (COPD [n = 1]; asthma [n = 2]). Among the 58 patients, 26 experienced pulmonary symptoms (age < 40 years [8/25]; ≥ 40 years [15/33]). Most patients described their symptoms as “breathlessness” (10/26) or “fatigue”/“fatigue due to limited endurance” (5/26). Daily functioning of 19/26 patients was limited due to the pulmonary problems and 18/26 patients reported worsening of symptoms with increased fatigue. Fourteen patients underwent scoliosis surgery and four of them experienced a relevant difference before and after the surgery. Although this survey undoubtedly has selection bias, these preliminary results indicate that pulmonary symptoms are prevalent and were commonly experienced in about half of both adolescent and adult patients.

## Continuous outcome monitoring: pulmonary function

In the era of value-based healthcare, to monitor the quality of the full cycle of care for patients with spinal disorders, routine outcome monitoring through outcome registries is valuable (Van Hooff et al. 2015). These outcome registries are based on a standard set of patient-relevant outcomes, i.e. patient-reported and clinician-based outcomes that matter to patients (Van Hooff et al. 2015). Based on the above, an outcome measure that includes patients’ experience regarding pulmonary function is clearly needed.

## Relevance of patient-reported outcome measure (PROMs): the way forward?

As yet, the theoretical construct of pulmonary function in both AIS and ASD, in terms of for example shortness of breath, and/or reduced exercise tolerance and/or respiratory fatigue, is not clearly understood. As such, no adequate methods are available that take patients’ perspective into account to quantify this in routine clinical practice. Clinical PFTs lack clinical relevance, as they do not cover the patients’ perspective, are time consuming, and are expensive to obtain in routine clinical daily practice. An adequate PROM might be a good alternative to assess pulmonary function in patients with spinal deformity. However, when following guidelines for the development of PROMs it takes several years to develop an adequate PROM in different languages and in terms of validity, reliability, and responsiveness (measurement properties). A general PROM development process, in which both clinicians and patients are involved, consists of several iterative steps that require mixed qualitative and quantitative (longitudinal) study designs (De Vet et al. 2014, Mokkink et al. 2019): definition of the theoretical construct to be measured, item generation, generating and selecting items, development of scales and scoring methods, initial pilot testing (feasibility and usability), and clinical field testing to evaluate its validity, reliability, and responsiveness (measurement properties). Our patient survey has demonstrated that we may indeed have been ignorant of an important aspect of adolescent and adult spinal deformity patients’ lives, and that we need to explore this further. Meanwhile, whilst work is performed to develop an adequate PROM, we recommend that care providers who see adolescents and adults with a spinal deformity should be aware of, pay attention to, and address pulmonary symptoms experienced, such as shortness of breath or reduced exercise tolerance.
